# Academy of Stomatology

**Published:** 1903-03

**Authors:** 

**Affiliations:** Academy of Stomatology


### ACADEMY OF STOMATOLOGY.

A stated meeting of the Philadelphia Academy of Stomatology was held at its rooms on Tuesday, October 28, 1902, at eight P.M., the President, Dr. R. Hamill D. Swing, in the chair.

A paper was read by Dr. J. G. Palmer, of New York, entitled "Conservatism *versus* Radicalism in Certain Dental Operations."

(For Dr. Palmer's paper, see page 171.)

### DISCUSSION.

*Dr. Louis Jack.*—I agree with nearly all that Dr. Palmer states, and will confine myself to a discussion of the well-worn subject of the treatment of pulpless teeth. For this purpose I will classify the cases under three headings.

1. Those cases with closed pulp-chambers and which when opened present no indication of effusion from the pericementum.

In these cases the canal may be empty and comparatively dry. The pulp may have been dead for years without noticeable pericemental disturbance. There may be no evidence of pus or exudate. It would seem a simple matter to disinfect and treat such cases, but there may be morbific matter present, which is liable to be aroused into activity.

I believe it best to proceed cautiously and to protect the canals from the entrance of oral fluids while treating. I pass the drill a little way only into the pulp-chamber, and then use a crystal potassium permanganate, followed by a drop of water. It is proper to then open up and obtain free access to the canals. These should then be sterilized, as far as possible, with sodium dioxide or hydrogen dioxide. This done, the roots may be reamed out with a Gates-Glidden drill and the dioxide again applied repeatedly.

Immediate filling might now be entirely successful but as occasionally serious disturbance arises I feel that it is safer to insert a solution of aristol in oil of cloves and to hermetically seal the external opening for a few days, when, if no trouble arises, the roots can be filled.

2. The second class of cases are those in which the pulp-chamber has been exposed to the oral fluids and hence the canals are infected by mouth germs. Generally there is pus in the apical region.

It is my practice in these cases to regard the area of apical infection as indeterminate, so I disinfect and remove the canal contents as in the first class of cases, using only oxidizing agents and avoiding irritants, such as carbolic acid. For the first regular dressing I again use a solution of aristol in oil of cloves, which liberates iodine to act upon the diseased apical tissue.

In cases of copious pus flow I insert a small broach in contact with a loose cotton dressing and pack gutta-percha about it into the external orifice. When the broach is withdrawn a vent is left. This proceeding is in accord with the surgical principle of drainage.

The first dressing, left for a day, determines the amount of possible pus collection at the apex. In healthy persons healing often results at once; in other cases it may persist. In the former case root-filling is in order; in the latter pus destruction and redressing is advocated.

If the foramen be closed, it is well to open it in any safe way, though this is often no easy matter. After opening, a zinc chloride solution, eight grains to one ounce of water, should be forced into the ulcer. This is easiest done in single-rooted teeth. In the multirooted the canals offer mechanical impediments, and the root abscessed is not always readily diagnosed.

3. In the third class of cases, in which a fistula exists, the zinc chloride solution is readily forced through it if the apex be opened. A mixture of carbolic acid and oil of cloves, equal parts, serves as well as the zinc chloride solution, and is less irritant than the weak solutions of carbolic acid.

Formalin in one or two per cent. strength may be substituted, but preferably for its diffusiveness, and should not be forced into non-fistulous abscesses. I would deprecate the leaving open of roots during treatment except when the small vent described is made for a short period in purulent cases. I also do not favor dallying treatment.

In cases of pulp removal after cocainization I would regard immediate filling as proper and also safe where the test by a solution of permanganate indicates the absence of organic matter.

In my belief the best root-filling is a paste of oxychloride of zinc.

*Dr. F. Milton Smith,* New York.—I can thoroughly agree with Dr. Palmer in all of his conservative ideas except those pertaining to immediate root-treatment. He quoted me as saying, in 1898, that I fill immediately almost without exception, but unfortunately placed an emphasis upon the wrong word. I do not deviate one iota from the strict interpretation of my statement, and I treat pulpless teeth, nearly without exception, but once, and that once thoroughly and radically. In infective cases I almost always open thoroughly under rubber dam, cleanse and disinfect the root-canal thoroughly with ninety-five per cent. carbolic acid, and force the acid through the root end into the infected area beyond the apical foramen, and then, before new infection occurs, I hermetically seal the root with chloropercha (in which iodoform is dissolved) on cotton. I make an exception in such rare cases as present the impossibility of obtaining complete dryness of the roots, owing to an excessive purulent collection in the apical space.

The simple discharge of pus which can be stopped at one sitting is no bar to my use of the immediate method. It is absolutely good practice to so proceed, and nature is fully competent to deal with any material remaining after thorough stimulation of the apical tissue. I also do not except the cases of dry canals with offensive odor. I rarely remove pulps surgically or in a non-infective condition, but would as readily except such a case as any infected one.

I treated a central incisor containing a dead pulp of the dry variety in 1891 by the immediate method, and after the second day the tooth was comfortable. It so remains to this day.

As a rule, I suggest to my patients the probability of a certain amount of healing inflammation after following my treatment, and if it is more than a passing irritation I apply the strongest tincture of iodine in a radical manner to the gums over the apex, and it accomplishes wonders as a counterirritant. The exceedingly painful cases are rare in my practice.

This treatment, so far from giving me more trouble than the conservative method, has the reverse result. I formerly treated conservatively, and then had occasionally to get out of bed or to lose my meals to relieve cases that had been filled only after several treatments. I regret to disagree with Drs. Palmer and Jack, but am compelled to do so.

I have never endorsed lancing for trouble after immediate root-filling. In a given case Dr. Palmer sought my advice for a swelling and suppuration he had had after immediate root-filling, and I then advised the use of a lancet.

As to the amount of trouble following my method, I would say that with a moderately full practice I have not lost ten teeth in thirteen years, and two of these had had arsenic applied repeatedly and in one case at least the root had been only filled half-way to the apex. Given careful and thorough opening and cleansing, thorough antisepsis of root and apical tissue, and thorough antiseptic root-filling, I believe that any careful operator who treats and fills immediately will succeed far beyond the one who removes two-thirds of the canal contents and after awakening the bacteria in the remaining third trusts to his antiseptic to devitalize them gradually. To do the latter is to invite the trouble we seek to avoid. We should rather kill them at once in a radical way before they have time to do injury.

I should like to discuss the other subjects touched upon by Dr. Palmer, as they are full of interest, but I have fully consumed the time allotted to me.

*Dr. Frank T. Gardiner.*—Having practised extension for over twenty years, the good results of its use in the teeth of my discriminating patients compel me to unhesitatingly advocate it for all proximal and occlusal cavities in bicuspids and molars. Originally educated to a method the reverse of this in principle, I was driven to extension because of the persistent failure of my most painstaking operations, where the margins were left in contact with the adjoining teeth. When I afterwards extended the margin beyond the contact point my failures ceased in all cases where I was able to perform satisfactory work. It is a principle that will prevail because correct for teeth liable to recurrence of caries. For those practically immune it is not necessary, though it must not be forgotten that a period of pronounced decay may supervene at some future time.

Dr. Black has clearly expressed the point that caries occurs in greatest intensity at a spot near the contact point, and that the influence of its cause decreases in a widening zone from this centre until the immune area is reached. This fact has been overlooked by the editor of the *Items of Interest* in his editorial point as quoted by our essayist, for if the margin of the filling remain within this zone caries at the margin is quite explainable as a result more natural than the formation of a new and independent cavity. In my experience such fillings in mouths in which decay is persistent are comparatively soon doomed to failure in the majority of cases, even though inserted as skilfully and perfectly as possible.

I do not practise extension, and do not approve of it in cases of small cavities in anterior teeth which involve only the points of contact, as to do this involves the inexcusable sacrifice of large portions of sound tooth-structure. I do not use gold in these cases if the teeth are prone to decay. I, however, practise it in all proximal cavities in anterior teeth that are larger than the smallest class if gold is to be inserted.

When I say that I extend cavities on the approximal surfaces of the oral teeth, I do not wish to be understood as extending them well out on the labial wall, so "as to show distinctly," as advocated by Dr. C. N. Johnson and, I believe, also by Dr. Black.

This practice is most inartistic, highly disfiguring, and altogether unnecessary.

My experience would indicate that the point of contact is the centre from which to extend, and that the protection of the tooth does not always require that the gingival margin shall be carried well above the gum.

In young patients the gingival margin would always be carried above the gum margin, for the reason that in the young mouth the whole interproximal space is completely filled with gum tissue to the point of contact. In adult life, as we all know, the position of the gum margin is often far removed from the contact point. When this condition exists it is not necessary to extend above the gum margin.

Have not some of us been hypercritical in these matters and seeking for possible objection rather than for the good that may possibly be in them? I believe all the radical methods have their merits, and should be practised with judgment and discretion when the best interests of the patient can be served by them.

*Dr. S. H. Guilford.*—In regard to the matter of immediate root-filling I do not understand the question as interpreted by Dr. Jack. I never understood it to mean the removal of a mass of devitalized pulp, disinfection, and filling of the canal, but that it meant the devitalization of the pulp and its removal, or at least removal of the pulp followed by immediate filling of the canal. When we remove a pulp which is in a purely physiological condition, as, for example, for crown- or bridge-work, there is no objection to the filling of the canal at once, because it becomes nothing more than an aseptic surgical operation. On the other hand, when in former years we found a pulp dead, and undertook to remove it, in spite of our utmost care we would often have trouble. Under such circumstances I do not understand why Dr. Smith does not have bad results. He says that whenever he fills such a tooth he expects trouble and tells the patient so, and the patient finds he is correct. The patient calls him up on the telephone. Dr. Smith makes a positive statement to him that the pain will be gone "to-morrow." He must be successful, or he would not have as many patients as he has. I do not understand why he should be so successful in this line where others have failed. The question arises, Is the practice he advocates a good one for others to follow? Does it carry within itself the elements of reason that will commend it as a safe line to follow?

I believe in a certain amount of conservatism, and therefore I agree with Dr. Palmer that where there has been a pathological condition it is better to diagnose the case correctly. When Dr. Smith has the root filled he has not the same advantage as when the root is simply dressed. I do not mean by this that careless work should be done, but that careful, modern treatment should be used with the rubber dam to get the root in as aseptic a condition as possible. When I was a student the method of pumping carbolic acid through the tooth was considered very thorough treatment. To-day, as a rule, no one considers carbolic acid. There are other remedies that will do the work a great deal better.

Our friends in the western part of the country tell us that in every case of crowning the tooth must be devitalized because it is tender and will give subsequent trouble. I have been setting crowns ever since the custom was introduced and I have never had any serious results that I can recall. The difference seems to lie in the fact that the men who are radical pursue radical methods throughout. They grind teeth pretty freely, and, of course, have trouble. We can take sufficient from the tooth to answer the purpose of placing a crown without cutting away half the substance of the tooth. I utterly condemn the method of devitalizing the pulp in every case before placing a crown. I do not believe I ever devitalized a tooth for the simple purpose of putting a hollow metal crown on it.

*Dr. J. B. Locherty,* New York.—I have always felt that I could get better adaptation of a crown by removing the pulp. In instances in which I have placed crowns without devitalization of the pulp, pulp-stones have formed. I believe that most of us will agree that a tooth is less liable to pyorrhœa when the pulp is devitalized. Broadly speaking, there probably is a little too much radicalism, especially among the dentists of the younger school.

In regard to extension for prevention or cutting of the tooth in a radical manner, I think more care should be devoted to the preparation of the cavity than to the general line of extension for prevention.

My practice in filling children's teeth is to use some plastic material, believing that gold filling is a too radical treatment for them.

*Dr. H. C. Register.*—I think dentists, as a rule, place too much dependence upon mechanical manipulations. We must get down to the principle involved and treat the disease from the present stand-point of its cause. In 1886 I commenced dehydration of the roots of teeth, and by its use dentine is made capable of absorbing germicides, and can thus be made absolutely sterile. I dehydrate by means of hot compressed air under fifteen to twenty-five pounds pressure. The heat should be as great as the patient can bear. The mouths of the tubuli are thus opened so that the fibrillæ can be acted upon. It makes no difference what germicide is used, provided it is not severe enough to cause irritation of the pericementum.

I think it ridiculous to say that we should fill children's teeth with gold. I have noticed almost invariably but little acidity in the mouths of children, while almost always you will find in the adult mouth that the saliva is acid. When we act from an understanding of the pathology of the conditions instead of depending upon mere mechanical work we will save hundreds of teeth which are being lost.

The operation of crown- and bridge-work is destroying as many teeth as it is preserving. As for extension for prevention, I think that the cases would be just as successful if extension were not carried quite as far as it is by some dentists. Gingivitis is usually the forerunner of all diseases of the roots outside of apical abscess and a very few other conditions. If dentists will take the trouble to use an electric lamp and acquire sufficient delicacy of touch in examination, they will find that nearly one-fourth of the patients that come to them have some form of gingivitis.

*Dr. Palmer.*—I confess I feel just a little bit pleased that I have not been sat upon any harder than I have. I knew my friend Dr. Smith came over here to combat me strongly, but I do not find that he has advanced any other arguments than he advanced in his paper to which I alluded. My impression is that he has overlooked one or two things in it to which I want to call his attention. My own experience in the treatment of devitalized teeth, extending over a longer period than Dr. Smith's, has always been very satisfactory. He spoke of losing some teeth; I tried to recall how many teeth I knew I had lost by reason of my treatment being unsuccessful, and was unable to recall more than two in my more than twenty-five years of practice, that have been extracted by reason of any difficulty in my method of treatment. There may be others which I do not know of. I have felt that I had some occasion to compliment myself upon my success. I have been particularly careful in the cleansing of canals, and have regarded thorough cleansing as being very important, whether I used a drug or not. I cannot understand why, if the immediate filling of the root-canal is the proper thing, there should be any necessity for pain. I am certain that by my own method I can treat the large majority of cases and not have pain. Dr. Smith and some others admit that they expect pain; that it is a common thing. Dr. Smith says that he gets his antiseptic through the root. I know that he tries to. I have a very pleasant acquaintance with Dr. Smith. I know of his success, but in this article of his to which allusion has been made he states that there are some very tortuous, minute canals that he does not get through. There he does the best he can, and lets it go at that. Probably those are the ones that do give him trouble.

Dr. Guilford spoke of waiting, after having removed the pulp, until the hemorrhage should stop, when the root can be filled. I speak in my paper of having been radical enough formerly to fill under such circumstances, but have had bad results occasionally, especially where there was profuse hemorrhage. I have had a good many cases in which I have not been able to stop the hemorrhage at one sitting, and it would continue for some time. When I have filled such canals without that hemorrhage ceasing I have always had trouble. After filling temporarily for two or three days with a proper dressing there will be no hemorrhage, and I can always fill.

I am glad that my other friend from New York, Dr. Locherty, agrees with me that there is too much radicalism, and especially regrets its effect upon the younger men. This was one of the special reasons why I wished to speak upon the subject. The younger man, in his desire to accomplish great things, takes up as a fad some of the methods employed by the older men, and, forgetting the details, does not succeed, thus bringing discredit upon the work.

DISCUSSION ON DR. T. VICTOR SMITH'S PAPER, "PORCELAIN INLAYS."

*Dr. L. Foster Jack.*—In speaking of the deep cavities in which the proper cement had not been used, Dr. Smith spoke of the necessity of going to the extra trouble of using a pin. I think in the case of the incisor teeth it is well used, but in the first case he spoke of I would deem it entirely unnecessary. I have on occasions attempted to remove ordinary inlays, not having succeeded in obtaining the proper color, and find them most difficult to remove. In one instance I used a diamond drill before the cement would give way.

*Dr. H. B. Hickman.*—I would like to ask Dr. Smith if the inlay he speaks of is a glass. If so, how long has it been in use, and will it turn black as all other glass has done?

*Dr. Smith.*—The material is No. 20 of Brewster's bodies, and is a porcelain, but of probably lighter color than any of the others. It has only been in use about six months. Dr. Reeves has used it a short time before it was put on the market. I cannot tell whether it will change color. It is, however, a porcelain, and should give perfect satisfaction. It is probably of somewhat lighter color than No. 1 enamel body shade. It is not entirely transparent. A considerable amount of it would change the color, and it is therefore only used in very small amounts. A porcelain is a chemical combination of two or more non-fusible substances in a fusible matrix, while glass is a combination of two or more fusible silicates.

(Replying to Dr. Jack.) There are some teeth, particularly the laterals, in which with strong occlusion it would be difficult to obtain sufficient anchorage without the use of a pin extending into the root-canal, and, of course, in those cases I deem it necessary to use it. There are a great number of cases where the pulp-chamber itself can be used and sufficient bulk to the inlay secured to give adequate anchorage. There are, however, many cases in which we need more anchorage.

On motion of Dr. E. T. Darby, a vote of thanks was extended to the essayists of the evening.

Adjourned.

OTTO E. INGLIS,
*Editor Academy of Stomatology.*

